# CDT1 is a Potential Therapeutic Target for the Progression of NAFLD to HCC and the Exacerbation of Cancer

**DOI:** 10.2174/0113892029313473240919105819

**Published:** 2024-10-04

**Authors:** Xingyu He, Jun Ma, Xue Yan, Xiangyu Yang, Ping Wang, Lijie Zhang, Na Li, Zheng Shi

**Affiliations:** 1 Clinical Medical College & Affiliated Hospital of Chengdu University, Chengdu University, 610083, P.R. China;; 2 West China Hospital, Sichuan University, 610083, P.R. China

**Keywords:** Nonalcoholic fatty liver disease, hepatocellular carcinoma, machine learning, CDT1, single-cell sequencing, early diagnosis

## Abstract

**Aims:**

This study aimed to identify potential therapeutic targets in the progression from non-alcoholic fatty liver disease (NAFLD) to hepatocellular carcinoma (HCC), with a focus on genes that could influence disease development and progression.

**Background:**

Hepatocellular carcinoma, significantly driven by non-alcoholic fatty liver disease, represents a major global health challenge due to late-stage diagnosis and limited treatment options. This study utilized bioinformatics to analyze data from GEO and TCGA, aiming to uncover molecular biomarkers that bridge NAFLD to HCC. Through identifying critical genes and pathways, our research seeks to advance early detection and develop targeted therapies, potentially improving prognosis and personalizing treatment for NAFLD-HCC patients.

**Objectives:**

Identify key genes that differ between NAFLD and HCC; Analyze these genes to understand their roles in disease progression; Validate the functions of these genes in NAFLD to HCC transition.

**Methods:**

Initially, we identified a set of genes differentially expressed in both NAFLD and HCC using second-generation sequencing data from the GEO and TCGA databases. We then employed a Cox proportional hazards model and a Lasso regression model, applying machine learning techniques to the large sample data from TCGA. This approach was used to screen for key disease-related genes, and an external dataset was utilized for model validation. Additionally, pseudo-temporal sequencing analysis of single-cell sequencing data was performed to further examine the variations in these genes in NAFLD and HCC.

**Results:**

The machine learning analysis identified IGSF3, CENPW, CDT1, and CDC6 as key genes. Furthermore, constructing a machine learning model for CDT1 revealed it to be the most critical gene, with model validation yielding an ROC value greater than 0.80. The single-cell sequencing data analysis confirmed significant variations in the four predicted key genes between the NAFLD and HCC groups.

**Conclusion:**

Our study underscores the pivotal role of CDT1 in the progression from NAFLD to HCC. This finding opens new avenues for early diagnosis and targeted therapy of HCC, highlighting CDT1 as a potential therapeutic target.

## 1 INTRODUCTION

Hepatocellular carcinoma (HCC) is estimated to be one of the top four causes of cancer-related deaths worldwide [1]. Non-alcoholic fatty liver disease (NAFLD) is the most common liver disorder and is attributed to many cases of HCC in numerous developed countries [2, 3]. Mechanisms of NAFLD include insulin resistance and oxidant stress, which leads to simple adipose lesions, chronic inflammation, and cellular fibrosis ultimately leading to a pro-cancer state in HCC [4, 5]. Studies have also reported that immunoregulatory mechanisms contribute to the disease outcome in the progression from NAFLD to fatty liver disease [6]. NAFLD can synergize with other risk factors such as metabolic dysfunction, chronic inflammation, genetic mutations, and immune dysregulation, accelerating the development of HCC [7]. Compared to viral HCC, patients with NAFLD-HCC are often challenging to detect in the early stages, frequently being diagnosed at an advanced stage of the pathology [8]. Moreover, many NAFLD-HCC patients face limitations in pharmacotherapy due to hepatic dysfunction induced by liver cirrhosis and metabolic syndrome-associated pathologies. Consequently, surgical resection remains the primary choice for HCC treatment for most patients, as recommended by local and global clinical guidelines [9]. Therefore, early diagnosis and prevention of the development of HCC emerge as the most effective strategies for improving patient prognosis, especially for those with NAFLD [10]. The screening and identification of gene functions are key steps in molecular biology and translational medicine research, helping us to understand the pathogenesis of diseases and guide the formulation of clinical treatment strategies. In this study, we collected a large amount of expression data from databases such as Gene Expression Omnibus (GEO) and The Cancer Genome Atlas (TCGA). Through a series of bioinformatics analyses, we identified molecular biomarkers related to the transition from NAFLD to HCC. The identification of new potential marker genes for risk assessment and targeted treatment may help improve patient survival rates and treatment outcomes.

The liver has a remarkable regenerative capacity as an adaptive response to continuous damage [11]. However, in the context of NAFLD, the repeated damage and regeneration processes occurring in chronic liver diseases (such as hepatitis, fatty liver disease, *etc*.) may lead to a dysregulation of cell proliferation [12]. Abnormal proliferation of liver cells not only increases the risk of genetic material errors but may also promote the activation of tumor promoters and the inactivation of tumor suppressors, thereby creating conditions for the occurrence of HCC [13]. Moreover, the chronic inflammatory environment serves as a promoting factor for carcinogenesis, further driving abnormal liver cell proliferation and tumor development by activating liver fibrosis and hepatic stellate cells [14]. Therefore, gaining a comprehensive understanding of the molecular mechanisms of abnormal liver cell division and proliferation in NAFLD, especially the intervention possibilities in this process by regulating the cell cycle, suppressing inflammatory responses, and preventing fibrosis progression, is crucial for developing strategies to prevent HCC [15, 16]. This requires not only focusing on the abnormal lipid metabolism of the liver but also on the precise regulatory mechanisms of cell proliferation during liver regeneration and how to maintain this balance in chronic liver diseases [17].

In conclusion, the objective of this study is to delineate the molecular landscape of the transition from NAFLD to HCC through multidimensional bioinformatics analysis, from the perspective of gene expression. By employing this approach, we aim to offer new insights and strategies for the early diagnosis and treatment of NAFLD-HCC and to provide a solid scientific foundation for personalized medicine for NAFLD, HCC, and related diseases. By identifying new key genes and screening potential drugs, we hope to contribute to improving patient prognosis and enhancing treatment outcomes.

## 2 MATERIALS AND METHODS

### 2.1 Data Acquisition and Filtering

We conducted a search for gene expression profiles and related documentation of patients with NAFLD in the Gene Expression Omnibus (GEO) database (http://www.ncbi. nlm.nih.gov/geo/) using the term “nonalcoholic fatty liver disease.” The inclusion criteria were as follows: 1. The data included a control comparison between fatty liver groups and healthy groups. 2. All samples originated from human liver tissue. 3. The dataset's raw data or gene expression profiles were availabled in GEO. Following these criteria, we identified the NAFLD-related dataset (GSE207310, GSE213621, GSE193066) [18-20]. Additionally, data on HCC were obtained from The Cancer Genome Atlas (TCGA) (https://portal.gdc.cancer.gov/) database, selecting the TCGA-LIHC cohort. This cohort (LIRI-JP) includes RNA sequencing data and corresponding clinical information for 369 HCC samples. For validation datasets, a cohort of 68 HCC samples was selected from the International Cancer Genome Consortium(ICGC) database (https://dcc.icgc.org/). Furthermore, single-cell data from liver biopsy samples of 3 patients who had both NAFLD and HCC were selected from the GEO database (GSE189175) [21].

### 2.2 Identification of Co-expressed Differential Genes

First, we utilized the DESeq2 package [22] in RStudio software (version 4.2.3) to perform differential analysis on genes between the disease and control groups within the NAFLD dataset and the TCGA-LIHC dataset. The criteria for identifying differentially expressed genes (DEGs) were set as |Log2Fold Change| > 1 and |adj.*P* Value| < 0.01 to identify differentially expressed genes (DEGs). Subsequently, we employed the 'ggplot2' package (https://ggplot2.tidyverse.org) to visualize the results of the differential analysis through volcano plots. Finally, we used the 'ggvenn' package (https://github.com/yanlinlin82/ggvenn) to integrate and identify the co-expressed DEGs between the NAFLD dataset and the HCC dataset.

### 2.3 Functional and Pathway Enrichment Analysis of Differential Genes

To explore differentially expressed genes co-expressed in both diseases, we used the cluster profile package in RStudio (version 4.2.3) to perform Gene Ontology (GO) terms and GSEA enrichment analysis. A *P*-value < 0.05 was set as the cutoff threshold, and the Benjamini-Hochberg method was employed to perform multiple corrections on the raw *p*-values calculated during functional enrichment analysis [23, 24]. Additionally, we utilized other packages, including dplyr, org.Hs.eg.db (https://bioconductor.org/packages/org.Hs.eg.db/) and enrichplot. These packages are instrumental in gene annotation, database connections, and the conversion of gene identifiers to gene names [25, 26].

### 2.4 Prognostic Model Construction through Machine Learning

Before model construction, cases with a survival time of less than 30 days were excluded from both the TCGA-LIHC and ICGC (LIRI-JP) datasets. The glmnet package in R was used to fit Cox proportional hazards models and Lasso regression models, with cross-validation applied to the Cox proportional hazards model to determine the optimal regularization parameter. Here, 1000 iterations of 5-fold cross- validation were performed to ultimately identify biomarkers from the common differentially expressed genes [27]. Then, using the 'survival' package, risk scores for each sample were calculated based on these five key genes, with the risk score formula: Risky score 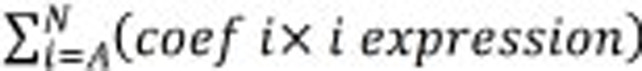 , where coef represents the model coefficient for each key gene, and expression represents the expression level of each key gene. The median of the risk scores was used as the threshold to divide samples into low-risk and high-risk groups. Survival analysis between the two risk groups and the generation of the receiver operator characteristic (ROC) curve were conducted using the survminer (https://rpkgs.datanovia.com/survminer) and timeROC packages [28].

### 2.5 Single-cell RNA-seq Integration Analysis and Pseudotime Analysis

Based on the single-cell dataset (GSE189175), which includes single-nucleus RNA-seq data from biopsy samples of both tumor and adjacent non-tumor tissues in patients with NAFLD and HCC, we performed principal component analysis (PCA) using the Seurat package in R [29]. Subsequently, the SingleR package (https://comphealth.ucsf.edu/app/singler) and CellMarker were employed to annotate the cells. The annotation results were validated based on the PCA findings [30]. Within the PCA and its annotation results, hepatocytes were selected for pseudotime analysis. The Monocle 3 package was used to cluster, learn, and order the cells, determining the root node and differentiation trajectory of cell differentiation to delineate the cell differentiation path [31].

### 2.6 Immune Infiltration Analysis

In his study, the CIBERSORT algorithm, an advanced computational method, was employed to quantify the extent of immune cell infiltration in samples from patients with HCC. The core advantage of CIBERSORT lies in its ability to estimate the relative abundance of 22 different types of immune cells from complex tissue expression data. This method relies on linear support vector regression (SVR) and uses gene expression signatures specific to each immune cell type, enabling accurate analysis of the composition of mixed cell populations [32]. We conducted a comprehensive analysis of each HCC patient sample, aiming to determine the extent of infiltration of different immune cells and their distribution proportions within the tumor microenvironment. Through CIBERSORT analysis, we successfully mapped the infiltration landscape of each type of immune cell in HCC, providing crucial insights into the role of immune cells in the progression of liver cancer. Additionally, this analysis revealed the complexity of the tumor immune microenvironment, laying the foundation for the development of future immunotherapy strategies [33].

## 3 RESULTS

### 3.1 Dataset Information

In this study, we selected the GEO dataset (GSE207310) and the TCGA-LIHC cohort data as the discovery cohorts. We selected the ICGC (LIRI-JP) cohort data, the NAFLD disease dataset (GSE213621, GSE193066), and the single- cell dataset (GSE189175) as external validation datasets. Detailed information about the data is presented in Table **1**.

### 3.2 Differential Gene Expression Analysis and Gene Set Enrichment Analysis (GSEA)

**Fig. (1):**
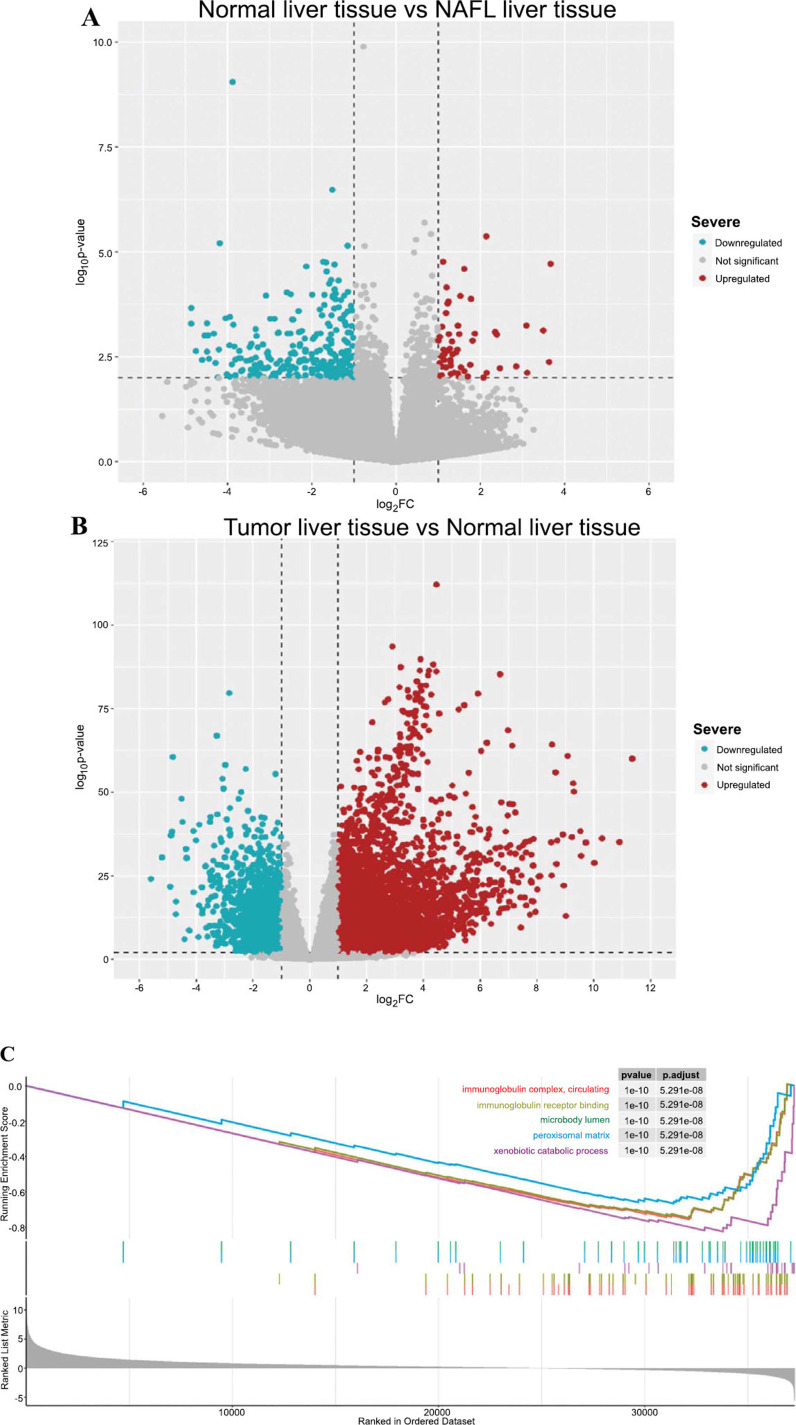
Differential Gene Expression and Pathway Enrichment in Liver Tissues. (**A**) Volcano plot illustrating differential gene expression between normal liver tissue and non-alcoholic fatty liver (NAFLD) tissue. (**B**) Volcano plot illustrating differential gene expression between tumor liver tissue and normal liver tissue (**C**) Gene Set En-richment Analysis (GSEA) plot.

In the analysis of the GEO dataset (GSE207310) comparing NAFLD cases with healthy controls, DEGs were identified in NAFLD patients, with 277 genes upregulated and 58 genes downregulated. These findings are represented in the form of a volcano plot (Fig. **1A**). In the TCGA dataset, significant differences in gene expression between HCC cases and healthy controls were found, with 6926 genes upregulated and 1802 genes downregulated, also represented as a volcano plot (Fig. **1B**). Using the GSEA method based on the TCGA database, we conducted further analysis on the differences between HCC and control groups. The top five ranked related pathways showed that DEGs in HCC are primarily related to the immunoglobulin complex, immunoglobulin receptor binding, and xenobiotic catabolic process (Fig. **1C**).

### 3.3 Enrichment Analysis of Common DEGs

**Fig. (2):**
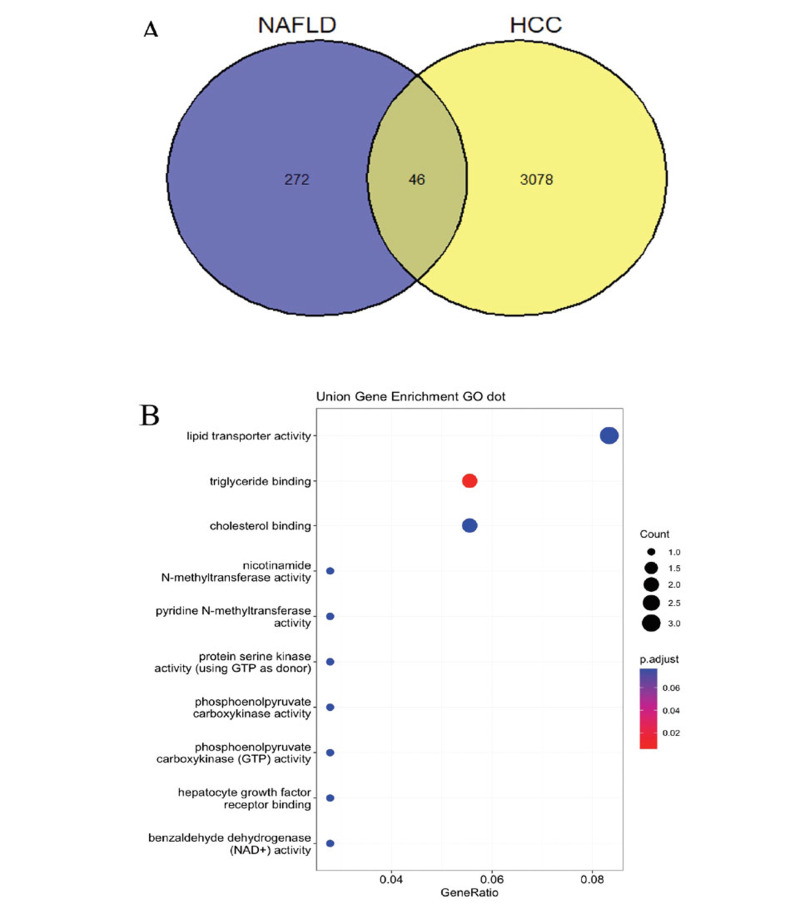
Overlap of Gene Expression and Functional Enrichment in NAFLD and HCC. (**A**) Venn diagram illustrating the overlap of differentially expressed genes between non-alcoholic fatty liver disease (NAFLD) and hepatocellular carcinoma (HCC). (**B**) Dot plot of Gene Ontology (GO) term enrichment analysis for the union set of differentially expressed genes in both NAFLD and HCC.

The Venn diagram illustrates that there are 46 common DEGs shared between 272 DEGs identified in NAFLD from the GEO dataset and 3078 DEGs identified in HCC from the TCGA dataset (Fig. **2A**). This study utilized the clusterProfiler package in R software to perform GO analysis to elucidate the roles and functions of these DEGs commonly expressed in both NAFLD and HCC. The GO enrichment analysis encompassed three aspects: Biological Processes (BP), Cellular Components (CC), and Molecular Functions (MF), with a focus on the 46 genes shared between both diseases. The results revealed that these genes are primarily involved in lipid transporter activity, triglyceride binding, and cholesterol binding (Fig. **2B**).

### 3.4 Construction and Evaluation of the HCC Prognostic Model

**Fig. (3):**
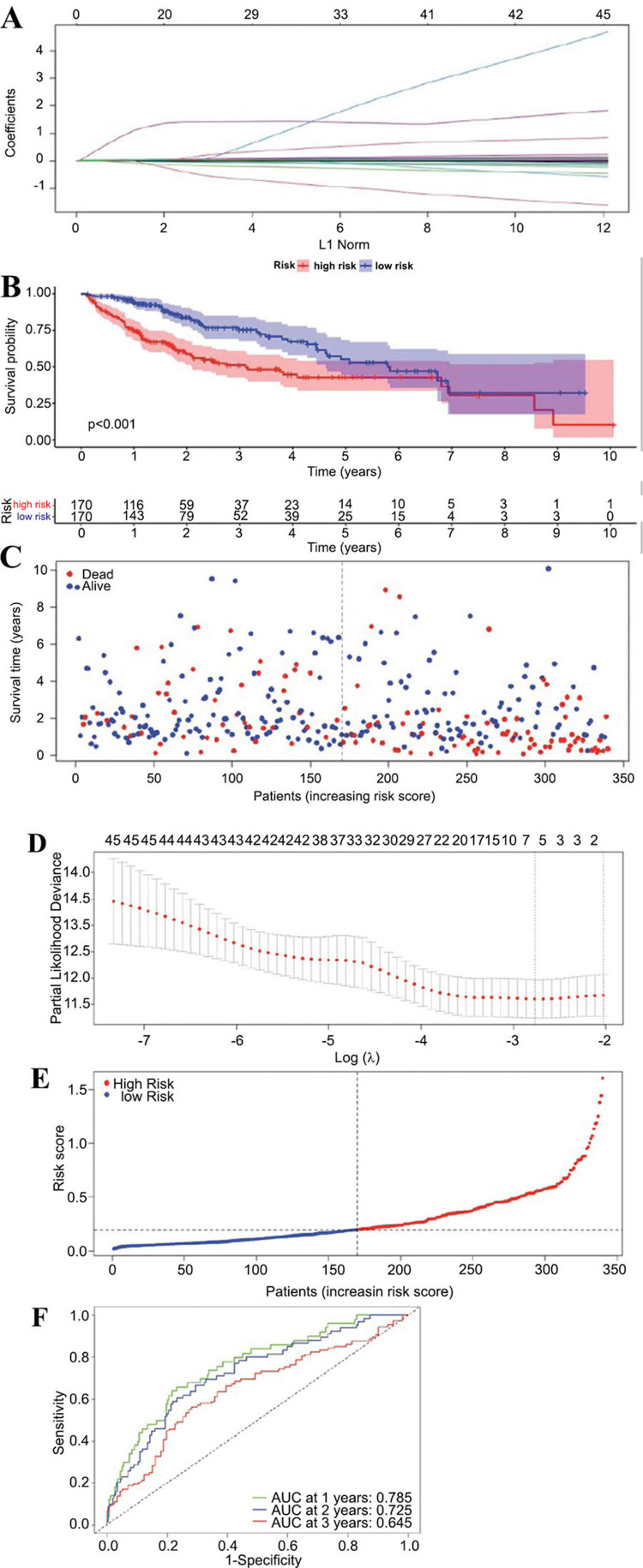
Construction of LASSO Cox prognostic model and evaluation of the HCC Prognostic Model. (**A**) Lasso coefficient profiles of the prognostic features across the log(lambda) sequence. (**B**) Kaplan-Meier survival curves stratified by high and low prognostic risk scores. (**C**) Scatter plot of survival status of patients against their prog-nostic risk score. (**D**) Tuning parameter (lambda) selection in the lasso model used in cross-validation *via* mini-mum criteria and the 1-standard error rule. (**E**) Risk score distribution of patients. (**F**) Time-dependent Receiver Operating Characteristic (ROC) curves evaluating the predictive accuracy of the prognostic model at 1, 3, and 5 years.

Common DEGs from both NAFLD and TCGA samples were collected for further analysis. After excluding TCGA samples with a survival time of less than 30 days, 340 samples remained. The expression data of the 46 common DEGs in these samples were then used to predict the risk scores using a Lasso Cox linear model in conjunction with TCGA prognostic time. After 10,000 iterations of 5-fold cross-validation, based on the optimal λ, a subset of 5 genes from the common DEGs was identified: IGSF3, CENPW, CDT1, CDC6, C20orf144 (Fig. **3A**, **D**). The coefficients (coef values) corresponding to these 5 biomarkers are listed in Table **2**.

The risk score for each patient sample in TCGA was calculated based on the expression levels of the 5 genes, weighted by their respective coefficients. The calculation is as follows: Y(risk score)=(0.035×IGSF3)+(0.011×CENPW)+(0.002×CDT1)+(0.024×CDC6)+(0.411×C20orf144). Based on the median risk score, the TCGA training dataset (n=340) was divided into high-risk (n=170) and low-risk (n=170) groups. The Kaplan-Meier curve showed a significant difference in overall survival (OS) between the high-risk and low-risk groups, with patients in the high-risk group exhibiting a lower probability of survival and a worse prognosis (*P*<0.001, log-rank test) (Fig. **3B**). The linear distribution of the risk scores for HCC patients calculated by the model is shown in Fig. (**3E**), and the survival status of HCC patients is displayed in a scatter plot in Fig. (**3C**). The sensitivity and specificity of the prognostic model were evaluated using time-dependent ROC curves, and the area under the curve (AUC) was calculated. The results showed that the model's AUC values for predicting the OS of patients at 1 year, 3 years, and 5 years were 0.758, 0.725, and 0.645, respectively (Fig. **3F**).

### 3.5 External Dataset Validation of Model Predictive Capability

**Fig. (4):**
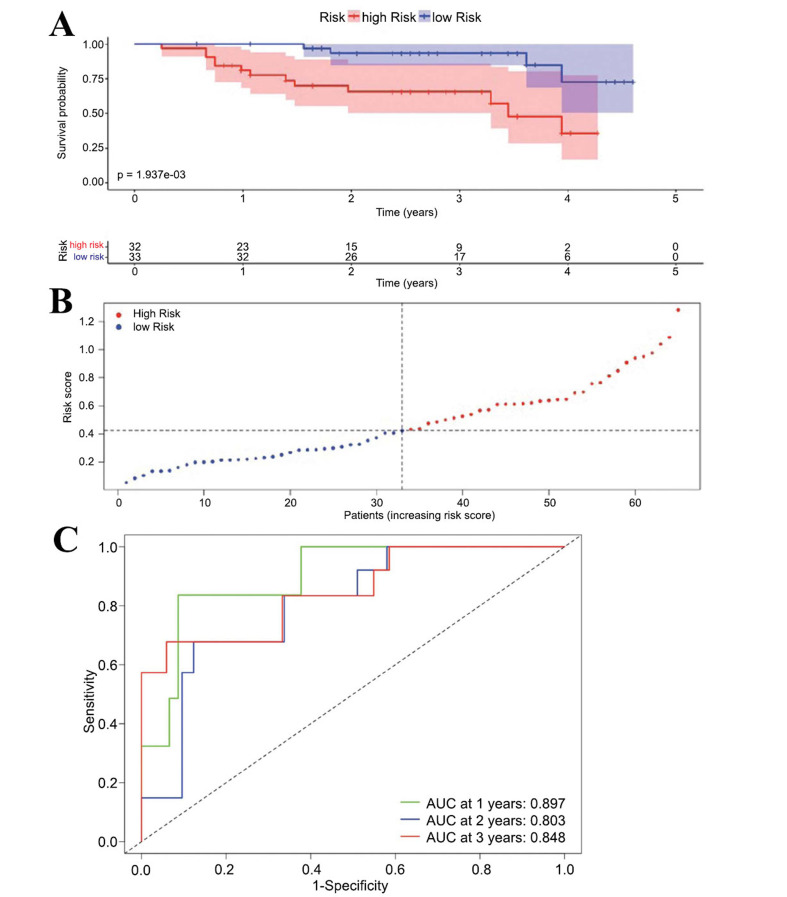
External Validation of the Prognostic Model's Survival Predictions and Risk Stratification Accuracy. (**A**) Kaplan-Meier survival curves for external validation cohorts, stratified by derived risk scores. (**B**) Risk score distribution among the external validation cohort. (**C**) Time-dependent ROC curves showing the performance of the prognostic model in the external cohort at 1, 2, and 3 years.

After constructing a prognostic model based on key genes, the predictive capability of the model was validated on the ICGC-JP dataset (n=65) for HCC patients. First, the risk score for each sample in the dataset was calculated. Based on the median risk score, the entire sample was divided into a high-risk group (n=32) and a low-risk group (n=33). Kaplan-Meier survival curves were plotted, revealing a significant difference in prognosis between the high and low-risk groups, with the high-risk group exhibiting a worse prognosis (Fig. **4A**). A linear graph depicting the distribution of risk scores among HCC patients in the external dataset was generated (Fig. **4B**). In the external dataset, ROC curve analysis showed that the AUC values of constructed model for predicting overall survival (OS) at 1 year, 3 years, and 5 years were 0.897, 0.803, and 0.848, respectively (Fig. **4C**).

### 3.6 Impact of Single Gene Expression Levels on Prognosis

**Fig. (5):**
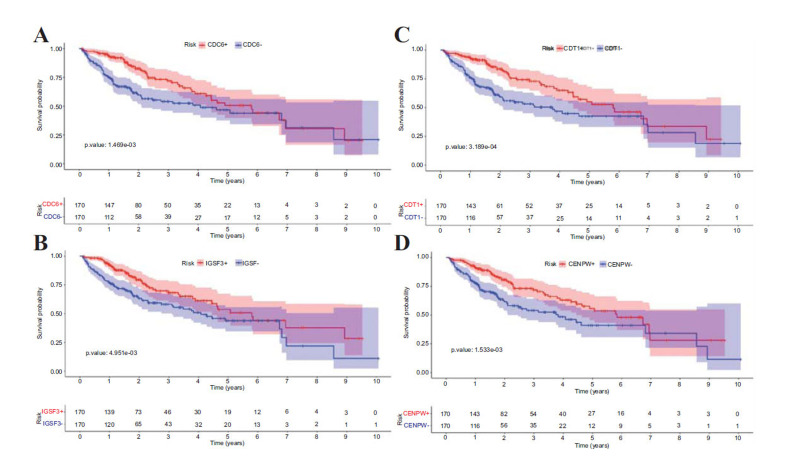
Single gene Survival Analysis Stratified by Biomarker Expression. (**A**) Kaplan-Meier curve comparing the survival probability over time between patients expressing CDC6 biomarkers. (**B**) Kaplan-Meier curve comparing the survival probability over time between patients expressing IGSF3 biomarkers. (**C**) Survival probability over time for patients categorized by CDT1 expression. (**D**) Kaplan-Meier curve showcasing the difference in survival between patients with CENPW expression. The accompanying risk tables and *p*-value are displayed below the curves.

Furthermore, this study investigated the relationship between the expression levels of each gene and the overall survival time of patients. Kaplan-Meier survival curves were generated for individual genes in relation to HCC patients. It was found that among the biomarkers, CDC6 (P.value=1.469e-03), IGSF3 (P.value=4.951e-03), CDT1 (P.value=3.189e-04), CENPW (P.value=1.533e-03) and CENPW (P.value=1.533e-03) exhibited a significant relationship between their expression levels and prognosis, with CDT1 exerting the most pronounced impact (Fig. **5A-D**).

### 3.7 Valuation Dataset for NAFLD from Bulk RNA-seq

**Fig. (6):**
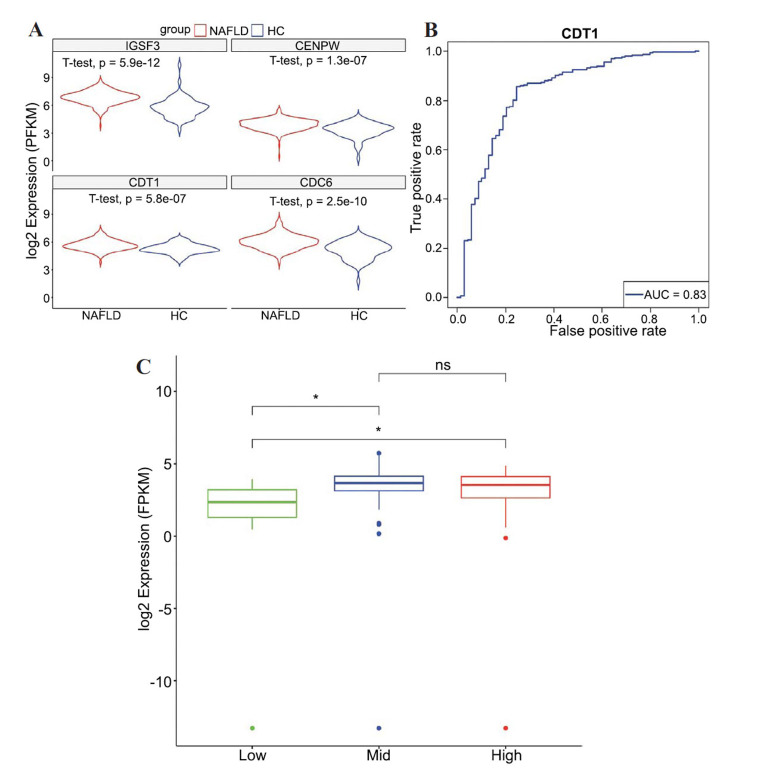
Gene Expression in Multiple GEO Datasets. (**A**) NFALD Liver Tissue Samples Comparing Normal. (**B**) Distinguishing Power of CDT1 Gene Between Normal and Disease. (**C**) Expression of CDT1 Gene Across Differ-ent NAFLD Activity Scores. *: 0.01<*p*<0.05.

In dataset, GSE213621, the expression levels of four key genes in NAFLD liver tissue samples were significantly higher in the NAFLD group compared to the normal group. These genes include IGSF3 (*P* = 5.9e-12), CENPW (*P* = 1.3e-07), CDT1 (*P* = 5.8e-07), and CDC6 (*P* = 2.5e-10) (Fig. **6A**). The AUC of gene expression used to distinguish between normal and diseased states for CDT1 is 0.85, indicating a high accuracy of CDT1 in distinguishing between normal and NAFLD groups (Fig. **6B**). In the dataset GSE193066, patients with NAFLD were grouped into high, medium and low groups based on activity scores assessed by pathologists^34^. The expression levels of the CDT1 gene increased significantly with higher NAFLD activity scores, suggesting that CDT1 could effectively predict the progression of NAFLD (Fig. **6C**).

### 3.8 Changes in Key Gene Expression Levels in Hepatocytes during the Transition from NAFLD to HCC

**Fig. (7):**
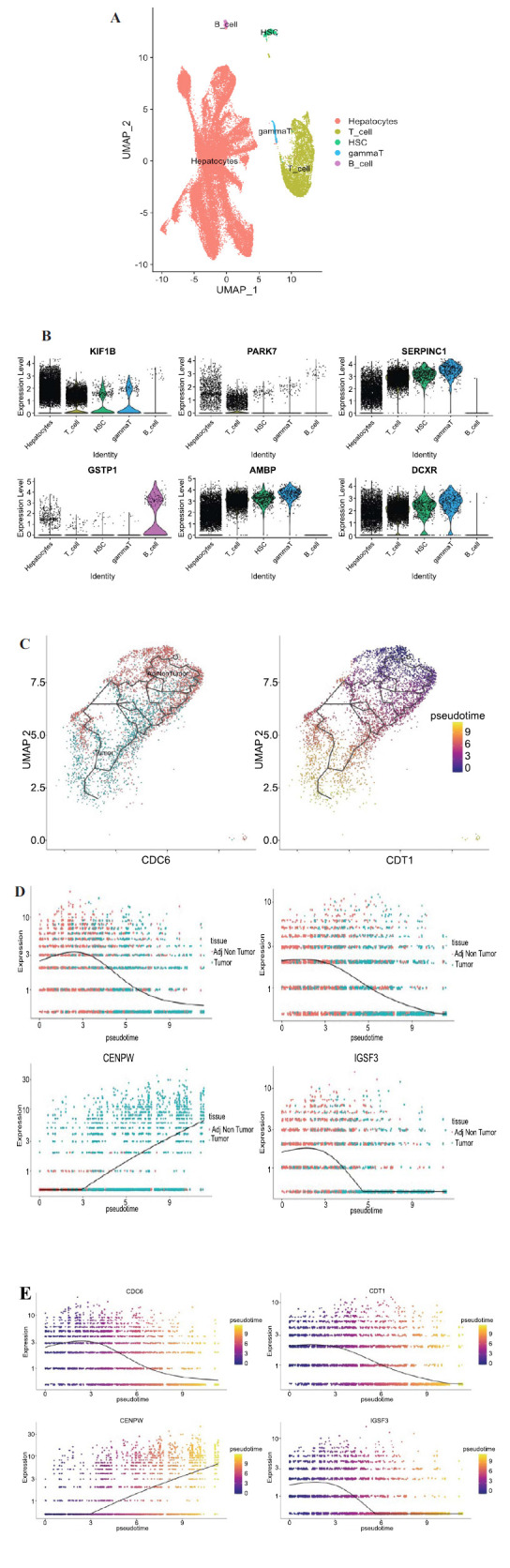
Single-Cell RNA-Seq Analysis of Liver Tissue from NAFLD and HCC Patients. (**A**) UMAP (Uniform Manifold Approximation and Projection) visualization of clustered cell populations in liver tissue. (**B**) Violin plots displaying the expression distribution of selected marker genes across different cell identities. (**C**) UMAP visuali-zation of single-cell RNA-seq data showing the pseudo-temporal progression of cell states from paraneoplastic cells to cancer cells. (**D**) Ridge plots depicting the expression patterns of genes associated with NAFLD and HCC across different cell types. (**E**) Feature plots showing the pseudo-temporal expression trajectory of the same genes as in Panel D.

Utilizing the single-cell dataset (GSE189175), we identified the top 2000 highly variable genes from both tumor cells and adjacent non-tumor cells. After conducting PCA, these genes were divided into 30 clusters. Based on the expression of lineage-specific markers, we identified distinct cell types, including hepatocytes, T cells, HSCs, gamma T cells, and B cells (Fig. **7A** and **B**). Hepatocytes were selected for single-cell sorting to delineate the differentiation trajectory nodes in adjacent non-cancer cells, resulting in a differentiation trajectory map and pseudo time plot from adjacent non-tumor cells to liver cancer cells (Fig. **7C**). Based on the results of single-cell pseudotime analysis, we examined the changes in the expression levels of the shared pathogenic genes (IGSF3, CENPW, CDC6, CDT1) from adjacent non-tumor cells to liver cancer cells during the continuous process. The pseudotime expression changes from a non-cancerous state to a liver cancer state were analyzed. The results showed differential expression levels of CDC6, CDT1, IGSF3, and CENPW (Fig. **7D** and **E**).

### 3.9 Immune Infiltration Characteristics in High-risk and Low-risk Patients

**Fig. (8):**
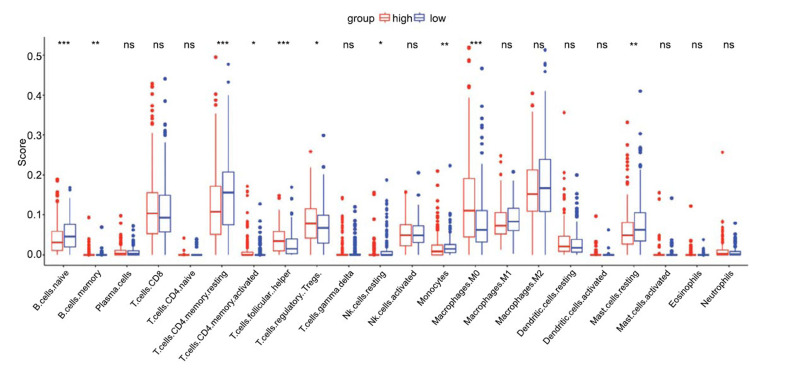
Immune cell infiltration score in high-risk *vs*. low-risk groups. This box plot displays the distribution of immune cell infiltration scores in high-risk (red) and low-risk (blue) patient groups.

In this study, CIBERSORT was employed to comprehensively assess the proportions of 22 types of immune cells in the two risk subgroups. The results showed that the infiltration scores of B cells and T cells (CD8+ and CD4+), as indicated by three asterisks, were significantly higher in the high-risk group (*P*-value <0.001). This suggests a potential link between the co-expressed genes associated with the progression from NAFLD to HCC and an active immune response. NK cells and dendritic cells (DCs), which exhibited significantly higher infiltration scores in the high-risk group, may reflect an enhanced response of the immune system to the aggressive tumor biology associated with the high-risk gene expression profile. The infiltration levels of monocytes and macrophages (M0, M1, M2) showed no significant differences, indicating that the involvement of these immune cells may not be affected by gene expression risk stratification or may represent a common feature of the two risk groups. The results also noted the presence of variability and outliers, especially in NK cells and M2 macrophages, which could reflect individual differences in the tumor microenvironment or the potential complexity of immune-tumor interactions (Fig. **8**).

## 4 DISCUSSION

NAFLD is one of the leading factors contributing to the development and progression of HCC worldwide [7, 34, 35]. NAFLD can progress to Non-Alcoholic Steatohepatitis (NASH), with the malignant type NASH-HCC being one primary predisposing factor for HCC [36]. Studies also indicate that NAFLD serves as a significant risk factor for HCC, a cancer type intricately involving inflammatory and metabolic pathways [37]. The primary characteristic of NAFLD is the accumulation of fat within the liver, which may be related to various biological factors including metabolic abnormalities, gut microbiota imbalance, and inflammation [38-41]. Previously, the precise pathogenesis of NAFLD progressing to metabolic risk factor-related HCC remained incompletely understood. The advent of high-throughput gene chips and transcriptome sequencing has completely changed the systematic analysis approach to disease research [42]. RNA sequencing and high-throughput microarrays aid in identifying reliable biomarkers, classifying diseases, and revealing the mechanisms behind disease progression [43-45]. The discovery of new biomarkers helps in predicting risk and determining the most suitable treatment for individual patients [46, 47]. Therefore, investigating the changes in gene expression during the transition from NAFLD to HCC is a crucial biological process.

In this study, we focused on analyzing DEGs that are commonly expressed in both NAFLD and HCC. Through GO analysis, we identified 46 genes that are co-expressed in both NAFLD and HCC, showing significant enrichment in biological processes related to lipid transporter activity, triglyceride binding, and cholesterol binding. This finding underscores the critical role of lipid metabolism-related functions and pathways in the transition from NAFLD to HCC [48-50]. Abnormalities in lipid metabolism, such as the abnormal accumulation of fat in the liver and dysregulation of cholesterol metabolism, are commonly observed in this process [51, 52]. Genes involved in lipid transport, and triglyceride, and cholesterol binding may play an important role in regulating these lipid metabolic abnormalities. By participating in the transport and binding of lipids, these genes may influence the accumulation and metabolism of lipids in the liver, thereby contributing to the progression from NAFLD to HCC [53]. Furthermore, through GSEA, we found that DEGs associated with HCC are mainly related to the binding of immunoglobulin complexes, immunoglobulin receptors, and xenobiotic catabolic processes. This indicates that the occurrence and progression of HCC are closely related to the activity of the immune system and drug metabolism pathways. The involvement of immunoglobulin complexes and immunoglobulin receptors may play a pivotal role in the immune regulation and cell signaling mechanisms of liver cancer [54].

Through these analyses, we deepened our understanding of the roles of lipid metabolism and immune regulation mechanisms in the progression from NAFLD to HCC. Subsequently, the Lasso Cox linear model, in conjunction with TCGA prognostic time, was used to predict the risk scores of the samples. Four gene subsets were identified from the shared DEGs in both NAFLD and HCC, including IGSF3, CENPW, CDT1, and CDC6. The identification of these gene subsets provided robust clues for revealing co-expressed pathogenic genes. Single-cell pseudotime analysis results then identified co-expressed pathogenic genes for NAFLD-HCC, including IGSF3, CENPW, CDC6, and CDT1. CDC6 and CDT1 are proteins necessary for mitosis, IGSF3 is involved in regulating the function of immune cells, and CENPW is a gene associated with DNA replication [55-58]. The overexpression of CDC6 and CDT1 can lead to excessive DNA replication and related instability, thereby promoting the process of carcinogenesis [59]. The expression of IGSF3 and CENPW can affect the timing of homologous chromosome pairing, responding to anomalies within the cell genome [60, 61]. Therefore, further exploration into the biological significance triggered by the aberrant expression of these genes could provide groundbreaking insights into the onset and progression of liver cancer. CDT1, functioning as a key regulator of DNA replication licensing, plays an important role in the pathogenesis of HCC [62]. The overexpression of CDT1 in various cancers, including HCC, induces abnormal replication and malignant transformation [63]. Research has shown that CDT1 transcription is significantly upregulated in HCC samples compared to healthy liver tissue, and elevated CDT1 expression is associated with the progression of clinical stages and poor prognosis in HCC [64]. Recent studies have explored the development of small molecule inhibitors targeting the CDT1/Geminin protein complex. One such compound, AF615, blocks cell cycle progression and reduces cell survival in various cancer cell lines by mimicking the cellular effects of abnormal CDT1/Geminin expression. This compound selectively affects cancer cells with minimal impact on normal cells, highlighting its potential for cancer treatment targeting CDT1 [65].

In our research, through single-cell analysis technology, we delved into the liver tissues of patients with both NAFLD and HCC. We focused on the transition from peritumoral non-tumor cells to liver cancer cells, and we employed pseudotime analysis techniques to identify a series of key gene expression changes from the non-cancerous to the cancerous state of liver cells. Specifically, we observed a downregulation in the expression levels of the genes CDC6, CDT1, and IGSF3, while the expression of the CENPW gene was upregulated. These changes underscore the important roles these genes collectively play in the progression from fatty liver to liver cancer. CDC6 and CDT1 are key genes in DNA replication and cell cycle regulation [66, 67]. Their downregulation may disrupt the normal regulation of the DNA replication process and the cell cycle, creating conditions for the transition from NAFLD to HCC. IGSF3, a gene involved in cell adhesion and signal transduction, its downregulation may hinder normal cell communication, accelerating liver cell degeneration and tumor transformation [60]. On the other hand, the upregulation of CENPW might indicate abnormalities in chromosome separation and mitosis, associated with disordered proliferation and heightened aggressiveness of liver cells. The knockout of CENPW, combined with sorafenib treatment for HCC, exhibited significant effects [68]. This finding provides further evidence supporting the central role of cell cycle and DNA replication regulation in the development of liver cancer, while also highlighting the importance of cell communication in the exacerbation process of liver diseases.

The results of immune infiltration analysis suggest that the expression of hub genes defining high and low-risk groups in NAFLD-HCC may be related to the dynamics of the immune microenvironment. The gene expression profiles of the high-risk group indicate that the progression from NAFLD to HCC seems to be associated with an increase in immune cells. This may reflect immune surveillance mechanisms or an immune activation microenvironment conducive to tumor progression [69, 70]. The observed immune infiltration patterns, based on the expression of hub genes for classifying HCC patients into different risk groups, provide valuable insights into the immune status of HCC. The distinct immune characteristics associated with high-risk patients could impact the development of precision medicine approaches (including targeted immunotherapies) and serve as a foundation for further exploration of the immunobiology underlying the progression from NAFLD to HCC [71, 72].

Through the analysis of second-generation sequencing data and single-cell sequencing data, our study enhanced the comprehension of the dynamic gene expression changes during the progression from NAFLD to HCC. Moreover, we identified potential targets for future therapeutic strategies aimed at these key molecular pathways. Our research offers insights for further studies into the molecular mechanisms of liver cancer development and progression. However, it is important to note that our findings are predictive results derived from data mining and analysis, which require further experimental validation. Additional population studies and clinical research will help to confirm the association between these genes and the development of fatty liver and liver cancer, providing novel targets for treatment and prevention. We anticipate ongoing advancements in this research field, bringing forth new perspectives for the prevention and treatment of liver cancer.

## CONCLUSION

We utilized machine learning and single-cell RNA-seq analysis to investigate the relationship between NAFLD gene expression and the development of HCC. Ultimately, we identified 4 related hub genes (IGSF3, CENPW, CDT1, and CDC6). Subsequent analysis highlighted CDT1 as the gene most significantly associated with the prognosis of HCC. Additionally, single-cell RNA-seq analysis showed differences in gene expression levels of these hub genes during the progression from NAFLD to HCC.

## Figures and Tables

**Table 1:** Data characteristics of patients with NAFLD and HCC.

**Cohort**	**Sample Information**	**Disease**	**Tissue**
GSE207310	15 cases with NAFLD, 5 healthy controls	NAFLD	Liver
GSE213621	299 cases with NAFLD, 69 healthy controls	NAFLD	Liver
GSE193066	106 patients with different disease stages of NAFLD	NAFLD	Liver
GSE189175	3 cases with HCC and NAFLD	HCC&NAFLD	Liver
TCGA	377 cases with HCC	HCC	Liver
LIRI-JP	68 cases with HCC	HCC	Liver

**Abbreviations:** NAFLD: Non-alcoholic fatty liver disease; HCC: Hepatocellular carcinoma.

**Table 2:** Coefficients of prognostic genes in the predictive model.

**Gene ID**	**Coef**
IGSF3	0.035
CENPW	0.011
CDT1	0.002
CDC6	0.024
C20or144	0.411

## Data Availability

Gene Expression Omnibus (GEO): https://www. ncbi.nlm.nih.gov/geo/; The Cancer Genome Atlas (TCGA): https://portal.gdc.cancer.gov/; International Cancer Genome Consortium (ICGC): https://dcc.icgc.org/.

## LIST OF ABBREVIATIONS

DCs: Dendritic Cells
DEGs: Differentially Expressed Genes
GEO: Gene Expression Omnibus
GO: Gene Ontology
HCC: Hepatocellular Carcinoma
ICGC: International Cancer Genome Consortium
NAFLD: Non-alcoholic Fatty Liver Disease
NASH: Non-Alcoholic Steatohepatitis
OS: Overall Survival
PCA: Principal Component Analysis
ROC: Receiver Operator Characteristic
SVR: Support Vector Regression
TCGA: The Cancer Genome Atlas
